# Heat acclimation reduces the effects of whole-body hyperthermia on knee-extensor relaxation rate, but does not affect voluntary torque production

**DOI:** 10.1007/s00421-022-05127-7

**Published:** 2023-01-13

**Authors:** Ralph Joseph Frederick Hills Gordon, Jodie Natasha Moss, Federico Castelli, Thomas Reeve, Ceri Elen Diss, Christopher James Tyler, Neale Anthony Tillin

**Affiliations:** 1 School of Life and Health Life Sciences, University of Roehampton, Holybourne Avenue, London, SW15 4JD England, UK; 2 Faculty of Science and Engineering, School of Psychology & Sport Science, Anglia Ruskin University, East Road, Cambridge, CB1 1PT UK

**Keywords:** Contractile properties, Heat adaptation, Maximum voluntary contraction, Neural drive, Thermal strain, Rapid muscle contraction

## Abstract

**Purpose:**

This study investigated the effects of acute hyperthermia and heat acclimation (HA) on maximal and rapid voluntary torque production, and their neuromuscular determinants.

**Methods:**

Ten participants completed 10 days of isothermic HA (50 °C, 50% rh) and had their knee-extensor neuromuscular function assessed in normothermic and hyperthermic conditions, pre-, after 5 and after 10 days of HA. Electrically evoked twitch and octet (300 Hz) contractions were delivered at rest. Maximum voluntary torque (MVT), surface electromyography (EMG) normalised to maximal M-wave, and voluntary activation (VA) were assessed during brief maximal isometric voluntary contractions. Rate of torque development (RTD) and normalised EMG were measured during rapid voluntary contractions.

**Results:**

Acute hyperthermia reduced neural drive (EMG at MVT and during rapid voluntary contractions; *P* < 0.05), increased evoked torques (*P* < 0.05), and shortened contraction and relaxation rates (*P* < 0.05). HA lowered resting rectal temperature and heart rate after 10 days (*P* < 0.05), and increased sweating rate after 5 and 10 days (*P* < 0.05), no differences were observed between 5 and 10 days. The hyperthermia-induced reduction in twitch half-relaxation was attenuated after 5 and 10 days of HA, but there were no other effects on neuromuscular function either in normothermic or hyperthermic conditions.

**Conclusion:**

HA-induced favourable adaptations to the heat after 5 and 10 days of exposure, but there was no measurable benefit on voluntary neuromuscular function in normothermic or hyperthermic conditions. HA did reduce the hyperthermic-induced reduction in twitch half-relaxation time, which may benefit twitch force summation and thus help preserve voluntary torque in hot environmental conditions.

## Introduction

Hot environmental conditions impair exercise performance by raising thermal and physiological strain (Ely et al. 2007; Mohr et al. 2012; Morante and Brotherhood 2008; Périard et al. 2014a; Racinais et al. 2015). Repeated exposure to heat stress (heat acclimation (HA)) can lessen this strain (Guy et al. 2015; Chalmers et al. 2014; Tyler et al. 2016), by inducing beneficial adaptations to the heat (Taylor 2014; Sawka et al. 2011). These adaptations include a lower resting core temperature and heart rate (HR) and an increased sweat rate (Sawka et al. 2011). Short-term HA protocols (5 daily exposures) are practical and effective at inducing some of these changes (Garrett et al. 2011; Chalmers et al. 2014); however, ≥ 10 days are required for more complete adaptation (Sawka et al. 2011; Guy et al. 2015; Tyler et al. 2016). The thermoregulatory and cardiovascular responses to HA are well documented (Périard et al. 2015); nevertheless, comparatively little is known about the effect that HA might have on the neuromuscular system, or the time course of those effects. Knowledge of the shortest amount of time to see a benefit to neuromuscular function from HA may be of practical use.

Exercise in hot ambient conditions which leads to a rise in core temperature, can cause reductions in voluntary force, due to reduced neural drive to the muscle (Nybo and Nielsen 2001). A reduction in neural drive resulting in a reduction in voluntary force is also observed during passive heating models (without exercise) (Todd et al. 2005; Morrison et al. 2004; Thomas et al. 2006; Gordon et al. 2021). If the hyperthermia-induced reduction in neural drive could be attenuated, the consequences may be a preservation in voluntary force. Other physiological and thermoregulatory systems have been shown to adapt to the heat, improving exercise performance and capacity (Périard et al. 2015); it therefore seems plausible that neural drive may also adapt following HA. There are data to show 11-day passive HA has a protective effect on voluntary activation and torque production during sustained voluntary contractions in hot conditions (Racinais et al. 2017a), suggesting supraspinal adaptions to HA. Conversely, HA over 28 days using exercise-induced hyperthermia (1 h per day) has shown no protective effect on cognitive performance and fine motor tasks in the heat (Piil et al. 2019). Impairments to cognitive performance during heat stress are distinct to neuromuscular changes (Gaoua et al. 2011); therefore, further investigation on the influence of HA on neural drive is warranted.

Few studies have investigated the influence of HA on neuromuscular function, and their findings have been mixed. Following 5 days of active (exercise) HA, Wingfield et al. (2016) reported no change (high intensity active HA) or a decrease (low intensity active HA) in maximal voluntary torque (MVT) and no change in neural drive. In contrast, Osborne et al. (2021) observed increased MVT without an increase in neural drive, following 5 days of active heat acclimation, suggesting peripheral adaptation to the muscle. However, the active HA protocols used by Wingfield et al. (2016) and Osborne et al. (2021) make it difficult to isolate neuromuscular adaptations to HA from those adaptations to the active component of the HA. Removing the confounding influence of exercise and utilising passive heating, one study has shown benefits of HA on neural drive and MVT (Racinais et al. 2017a), and another none (Brazaitis and Skurvydas 2010). The discrepancy between the findings of Racinais et al. (2017a) and Brazaitis and Skurvydas (2010) is not clear, but may be due to differences in the adaptation stimulus (time at an elevated core temperature; Taylor 2014). In each study, HA was deemed successful [evidenced by reductions in rectal temperature (*T*_re_)], but the adaptation stimulus was lower in Brazaitis and Skurvydas (2010) (~ 20 min *T*_re_ ≥ 38.5 °C per session for 7 sessions) compared to Racinais et al. (2017a) (66 ± 8 min *T*_re_ ≥ 38.5 °C in 9/11 sessions), which might explain why only Racinais et al. (2017a) observed benefits of HA on neuromuscular function. Thus, a large thermal impulse may be required to induce neuromuscular adaptations, specifically to neural drive generation and transmission to the peripheral nervous system. Providing a large thermal stimulus (daily heating up to a *T*_re_ of 39 °C) may help to elucidate the effect of HA on neuromuscular function.

MVT is typically used to assess the capacity of the neuromuscular system. The relevance of MVT is reduced in functional situations, where time to develop torque is limited because MVT takes > 125 ms to achieve when contracting from rest (Tillin et al. 2012, 2018). An alternative assessment to MVT is measuring voluntary RTD, which quantifies the ability to rapidly produce torque throughout the rising torque–time curve from rest (Folland et al. 2014). The determinants of voluntary RTD derive from both central and peripheral mechanisms; however, the relative contributions of these pathways differ throughout the rising torque–time curve (Folland et al. 2014). Moreover, the effects of hyperthermia on voluntary RTD are less well known compared to MVT, with recent data showing there are distinct responses between the two variables in the heat (Gordon et al. 2021). Hyperthermia reduces neural drive at the plateau of an MVC (where MVT is measured). Gordon et al. (2021) also observed reductions in neural drive at the onset of rapid voluntary contractions. Interestingly, whilst these reductions in neural drive translated to reduced MVT, voluntary RTD remained unaffected by hyperthermia, despite neural drive at the onset of a rapid contraction being an important determinant of RTD (Folland et al. 2014; Del Vecchio et al. 2019). RTD appears to be preserved when hyperthermic due to faster intrinsic contractile properties (Gordon et al. 2021). Faster intrinsic contractile properties occur when muscles are warmer (de Ruiter et al. 1999; de Ruiter and de Haan 2000; Dewhurst et al. 2005). Theoretically, if a regime of HA with sufficient thermal stimulus can offer a protective effect on neural drive, in conjunction with the hyperthermia-induced faster intrinsic contractile properties, voluntary RTD may increase.

Independent of hyperthermia, increases in MVT have been observed in hot and cool conditions following 11 days of passive HA (Racinais et al. 2017b), without modifications to neural drive, suggesting HA may induce adaptations in baseline contractile properties. Evidence for this has been provided in rats by Kodesh and Horowitz (2010) who found increases in peak tetanic force after 30 days’ heat exposure. The same study (Kodesh and Horowitz 2010) also found decreases in the rate of relaxation after HA, suggesting HA modifies the re-uptake of Ca^2+^ by the sarcoplasmic reticulum. The slower relaxation rate following HA may have increased the tetanic force by enhancing twitch force summation, and a similar effect in humans may result in increased MVT. However, the effects of HA on the contractile properties and, in turn, voluntary torque production in humans remain unclear.

The aim of the current study was to investigate the effects of 5 and 10 days of HA on MVT, voluntary RTD and their neuromuscular determinants measured in normothermic and hyperthermic conditions. It was hypothesised that: (i) a regime of HA would attenuate the decline in neural drive caused by hyperthermia, attenuating any reduction in MVT and enabling an increase in voluntary RTD due to faster contractile properties; (ii) independent of hyperthermia, HA would reduce the relaxation time of muscle, benefiting evoked and voluntary torque output in normothermic and hyperthermic conditions; (iii) neuromuscular adaptations from HA would be more observable after 10, rather than 5 days of heat exposure, giving a time course of adaptation to the neuromuscular system following short- and medium-term HA.

## Methods

### Participants

Ten (5 females and 5 males) healthy, physically active individuals participated in the study. Mean age, body mass and stature were 35.6 ± 7.2 years; 70.7 ± 9.7 kg, and 175.7 ± 8.6 cm, respectively. An a priori power analysis was performed for sample size estimation (GPower 3.1) based on data from Gordon et al. (2021) (*n* = 9) comparing acute hyperthermia-induced decreases in EMG_MVT_ at T_re_ 39.5 °C compared to ~ 37 °C, and a large effect (0.14) using ${\eta }_{p}^{2}$. With an α = 0.05 and β = 0.80, the projected sample size needed for the present study to assess differences between baseline and hyperthermia was approximately *n* = 10. In addition, a very large effect size (Cohen’s *d* = 1.2; *n* = 8) was estimated from similar HA research using a 5-day protocol (Osborne et al. 2021) for the change in EMG between control and HA pre-cycling, at two time points; pre- and post-HA intervention. All participants were informed of any risks and discomforts associated with the experiment before giving their written informed consent, in accordance with the latest version of the Declaration of Helsinki. Experimental procedures were approved by the Ethical Advisory Committee of the University of Roehampton (LSC 19/259). Participants were considered non-heat acclimated because they had not been exposed to ambient temperatures exceeding 25 °C for the 3 weeks prior to participation. Participants were allowed to maintain their normal training routine during the HA days (*n* = 6 were training to compete in the ultra-endurance running foot race, Marathon des Sables, *n* = 3 were club-level runners and *n* = 1 a club-level rower). Participants were instructed to refrain from any strenuous physical activity for 24 h prior to visiting the laboratory for the experimental trials, and from alcohol consumption for the duration of the study. Due to the scheduling requirements of the study, it was not possible to control for the variations in hormone levels associated with the menstrual cycle for female participants. The authors recognise that this may have caused some variability in T_re_, contributing to some variability in neuromuscular function; however, ecological validity of the study was increased by not controlling for the menstrual cycle, as such control is not possible in real world/sporting scenarios. Furthermore, recent data suggest menstrual phase does not modulate whole body heat loss in hot conditions (Notley et al. 2019). Data were collected between March and May 2019 (mean outside temperature ~ 11 °C) at the University of Roehampton, London, in the UK.

### Overview

**Fig. 1:**
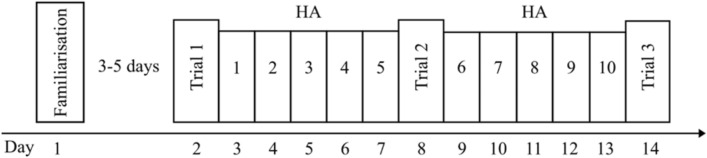
Schematic of the experimental study design

Participants visited the laboratory on 14 separate occasions, completing a familiarisation, three experimental trials, and 10 days of HA. The first experimental trial was completed 3–5 days after the familiarisation, with all remaining visits conducted on consecutive days (Fig. 1). All sessions were completed in a walk-in environmental chamber. The experimental trials and HA sessions were completed at the same time of day for each participant, and in the same ambient conditions (50 °C, 50% rh).

In the three experimental trials, participants completed two sets of the same neuromuscular protocol using their preferred leg as in the protocol of Gordon et al. (2021). The protocol comprised a series of involuntary and voluntary isometric contractions of the knee extensors. Set 1 was completed at a *T*_re_ of ~ 37 °C and set 2 at a *T*_re_ of ~ 39 °C.

The HA sessions employed an isothermic heat adaptation protocol to a target *T*_re_ of ~ 39 °C. There is no consensus on the optimal daily heat exposure, with a meta-analysis concluding research studies investigating HA have used session lengths with a mean duration of 105 ± 62 min (Tyler et al. 2016). To provide sufficient magnitude of thermal impulse (Taylor 2014) and maximum potential for thermal adaptation, we chose 90-min daily heat exposure up to a target *T*_re_ of 39 °C, which was adapted from previous research using a progressive protocol initially targeting a *T*_re_ of 38.5 °C, increasing to 39 °C (Gibson et al. 2015). After each session, participants were cooled in the laboratory (~ 21 °C) until *T*_re_ had returned to 38 °C.

### Protocol

#### Experimental trials

Skin thermistors and EMG electrodes were attached before the participants donned an impermeable rain jacket and trouser combination and entered the environmental chamber. Participants were seated in the isometric strength testing chair ready to complete set 1. To standardise the *T*_re_ during set 1 across experimental trials, and account for the expected reduction in resting *T*_re_ after HA (Tyler et al. 2016), set 1 was completed at a *T*_re_ of 37.0 ± 0.3 °C. If *T*_re_ was < 36.7 °C, participants remained seated in the strength chair wearing the jacket and trousers passively heating until *T*_re_ was within ~ 0.3 °C of 37.0 °C, and then set 1 commenced. If *T*_re_ was greater than 37.3 °C before entering the heat chamber, participants were asked to remain quietly seated in the ambient temperatures of the laboratory (~ 21 °C), until *T*_re_ had decreased to within ~ 0.3 °C of 37.0 °C. After completing neuromuscular set 1, participants performed a fixed intensity exercise bout (~ 80 W) on a cycle ergometer, to facilitate heat storage without eliciting exercise-induced fatigue, until a *T*_re_ of 38 °C was attained. Participants were then passively heated in either a seated or supine position, before transferring back to the isometric strength chair to perform set 2. The time spent passively heating ranged between 0–45 min (Trial 1), 5–58 min (Trial 2) and 5–60 min (Trial 3). To account for the expected rise in *T*_re_ but achieve a mean target T_re_ of ~ 39 °C during the neuromuscular protocol, set 2 commenced at 38.9 ± 0.1 °C in all trials. If participants had been resting in a supine position, sufficient time was allowed for a gradual change in posture and subsequent transfer back to the isometric strength chair. The impermeable rain jacket was worn continuously throughout the experimental trial. The trousers were removed to perform the neuromuscular sets, but were otherwise worn continuously.

#### HA sessions

Participants were instrumented with skin thermistors before entering the environmental chamber, sitting quietly on a cycle ergometer for 2 min, and having their baseline thermoregulatory and perceptual measurements recorded. A similar bout of light physical activity to that completed in the experimental trials was performed on the cycle ergometer. Initial work was 100 W, and this was subsequently reduced by 7 W every 5 min to 72 W. Participants cycled at 72 W until *T*_re_ reached 38 °C, then transferred to a chair located adjacent to the ergometer, and sat until *T*_re_ reached 39 °C. Upon reaching *T*_re_ 39 °C, participants removed the jacket and trousers and spent the remainder of the session (HA sessions were 90 min in total) supine or seated according to their preference. *T*_re_ was maintained as close as possible to the target 39 °C by donning and doffing the jacket and trouser combination, as necessary, based on real-time *T*_re_ data. To improve participant thermal comfort and facilitate the completion of the HA sessions, an electric fan was available if participants requested it for facial fanning only. This option was only provided during the HA sessions. Three participants were unable to complete one HA session each, due to personal circumstances. Overall, 98% of HA sessions were completed.

### Measurements

#### Torque

Seated in a custom-built strength testing chair (Maffiuletti et al. 2016), participants were securely fastened with a waist belt and shoulder straps with hip and knee angles fixed at 100° and 105°, respectively (180° defined full extension). An ankle strap, in series with a strain gauge load cell (FSB Universal Cell 1.5 kN, Force Logic, Reading, UK), was secured 4 cm proximal to the medial malleolus, with the load cell aligned perpendicular to the tibia during knee extension (Tillin et al. 2010). The force signal was amplified (× 375) and sampled at 2000 Hz via an analogue-to-digital converter (Mirco3 1401, CED, UK) and PC using Spike2 software (Spike 2 Version 8, CED, UK). A computer monitor, placed in view of the participant, provided real-time biofeedback. Off-line, the force signal was filtered using a fourth-order low-pass Butterworth filter with a 500 Hz cutoff frequency. To calculate knee extension torque, the weight of limb was corrected for and multiplied by the external moment arm (measured as the distance between the lateral knee joint space and the centre point of the ankle strap).

#### EMG

Following preparation of the skin (shaving, light abrasion, and cleaning using 70% ethanol) a bipolar silver–silver chloride gel-electrode configuration (2 cm diameter, and 2 cm inter-electrode distance; Dual Electrode, Noraxon, USA) was placed over the belly of the rectus femoris, vastus lateralis and vastus medialis. Electrode configurations were placed parallel to the presumed orientation of the muscle fibres at specific distances from the greater trochanter to the lateral knee joint space [44 ± 5% (rectus femoris), 71 ± 3% (vastus lateralis), 79 ± 3% (vastus medialis)]. The placement of electrodes was conducted by the same investigator in all trials and established during familiarisation. The position of each electrode was marked on the skin using permanent ink. Participants were instructed not to actively wash these marks off between trials, so electrodes could be placed in the same position at the beginning of each experimental trial. The EMG signal was amplified (× 500; 10–500 Hz bandwidth), transmitted wirelessly to a desktop receiver (TeleMYODTS, Noraxon, USA), and sampled (2000 Hz) in synchronisation with force via the same analogue-to-digital converter using Spike2 software. In off-line analysis, the EMG signals were band-pass-filtered between 6 and 500 Hz using a fourth-order Butterworth digital filter and time corrected (156 ms delay inherent in the TeleMyoDTS system) to align with the force signal.

### Electrical stimulation

Electrical square-wave pulses (200 µs duration) delivered over the femoral nerve (DS7AH Constant Current Stimulator, Digitimer, UK) were used to evoke twitch (single pulse), doublet (two pulses at 100 Hz) and octet (eight pulses at 300 Hz) contractions. The anode (rubber electrode 10 × 7 cm, EMS Physio Ltd, UK) was secured by tape (Transpore, 3 M, UK) to the skin over the greater trochanter. The cathode stimulation probe (1 cm diameter tip; S1 Compex Motor PointPen, Digitimer, UK), which protruded 2 cm from the centre of a custom-built plastic base (4 × 3 cm), was placed over the femoral nerve in the femoral triangle. The greatest evoked peak twitch force in response to a submaximal current determined the precise placement of the cathode, where it was taped in place. The electrical current was then increased incrementally by 20 mA until there was a plateau in both twitch peak force and peak-to-peak M-wave amplitude at each EMG site. This current was increased by a further 20% (supramaximal) to ensure all stimulations were eliciting a maximal involuntary response, and this current (122 ± 22 mA) was used for all twitch, doublet, and octet contractions thereafter. The cathode position and supramaximal stimulation intensity were determined for each participant in the familiarisation session and then kept constant for the experimental trials. The cathode position was marked on the skin with permanent ink and maintained by participants to ensure accurate relocation between trials.

### Thermoregulatory and perceptual responses

A rectal thermistor (REC-U-VL30, Grant Instruments, UK) was self-inserted ~ 10 cm past the anal sphincter to measure *T*_re_. Wireless skin thermistors (iButton DS1922L; Maxim/Dallas Semiconductor Corp., USA) were applied to the skin with a transparent dressing and secured with surgical tape for the assessment of local skin temperature. Mean weighted skin temperature ($\overline{T}$_sk_) was calculated from four skin sites located on the right side of the body: below the suprasternal notch (chest), flexi carpi radialis (arms), gastrocnemius (legs), and rectus femoris (thighs) (Ramanathan 1964). HR was recorded with a heart rate monitor secured with a strap and worn by the participant in contact with the skin (F3, Polar Electro, UK, Ltd).

Whole body thermal sensation (TS) was rated using a nine-point scale from 0 (unbearably cold) to 8 (unbearably hot) with 4 as comfortable (neutral) (Young et al. 1987). Whole body thermal comfort (TC) was measured using a four-point scale from 1 (comfortable) to 4 (very uncomfortable) (Gagge et al. 1967). All thermoregulatory and perceptual measurements were recorded at 5 min intervals and at the start and end of each of the neuromuscular assessment protocols in the experimental trials.

### Fluid loss

Participants consumed 500 ml of water 2 h before each visit to the laboratory. Pre-session hydration status was assessed from a mid-stream urine sample and euhydration was assumed if urine specific gravity was ≤ 1.020. Water (non-chilled) was provided ad libitum throughout and voluntary fluid consumption recorded. Participants were instructed to towel dry themselves, removing any residual sweat on the skin before recording nude body mass, pre- and post-sessions. After correcting for fluid intake and urine output, body mass changes were used to estimate sweat loss.

### Neuromuscular set

The series of involuntary and voluntary contractions used in the neuromuscular set in the experimental trials are described below, in the order that they were performed.

### Twitch and octet

Two electrically evoked twitch and octet contractions were delivered over the femoral nerve at rest, 20 s apart. The maximal M-wave (*M*_max_) was calculated as the average M-wave response (peak–peak amplitude of the EMG signal) from the two evoked twitches and used for EMG normalisation. Twitch and octet responses were analysed for: peak torque (PT); rate of torque development during the initial 50 ms from contraction onset (RTD_0-50_); peak rate of torque development (pRTD); time to peak torque (TPT); and half-relaxation time (½RT). Mean values were calculated for all dependant variables across the two twitch and two octet contractions.

### Rapid voluntary contractions

Participants performed 10–15 rapid contractions (~ 1 s) and were instructed to push as “fast and hard” as possible (Tillin et al. 2010), emphasising the “fast” element of the contraction. For each contraction, participants were encouraged to exceed 80% of MVT, as quickly as possible, repeating the effort if this was not achieved, up to a maximum of 15 contractions. Any rapid contractions with discernible countermovement or pre-tension prior to force onset were also repeated. A short recovery (5–10 s) was given between contractions and participants were instructed to relax their leg as quickly as possible before the next effort, which only commenced once force had returned to a stable baseline. Baseline force was displayed on a sensitive scale on a computer monitor in front of the participants to provide biofeedback on the occurrence of any countermovement or pre-tension. The slope of the force–time curve (25 ms time-constant) was also displayed. The three rapid voluntary contractions with the highest pRTD and no discernible countermovement or pre-tension (change in baseline force of > 2 SD of the mean during the 200 ms prior to force onset) were used for analysis. Torque was measured at discrete time points 50 ms (*T*_50_), 100 ms (*T*_100_) and 150 ms (*T*_150_) from torque onset, and RTD measured over three sequential time epochs: 0–50 ms (RTD_0-50_), 50–100 ms (RTD_50-100_) and 100–150 ms (RTD_100-150_). The RMS of the signal at each EMG site was assessed over 0–50 ms (EMG_0-50_), 0–100 ms (EMG_0-100_), and 0–150 ms (EMG_0-150_) from EMG onset, normalised to *M*_max_ at the same EMG site, and averaged across the three EMG sites to give a mean value for the quadriceps muscles. Dependant variables were mean averaged across the three rapid contractions selected for analysis. Torque and EMG signal onsets (voluntary and evoked) were identified using visual identification, which is considered the “gold standard” of signal onset determination compared to automated detection methods (Tillin et al. 2013), using the standardised protocol of Tillin et al. (2010). Mean values were averaged for the two twitch and octet contractions, respectively.

### MVCs

Participants performed three MVCs (3–5 s), separated by 30 s rest and were instructed to push as “hard” as possible. At the plateau of the second MVC, two superimposed involuntary doublet stimuli were evoked 2 s apart, followed by a doublet contraction evoked at rest 2–5 s after the MVC. MVT was defined as the greatest voluntary (i.e. not due to superimposed doublet stimulation) torque recorded in any of the rapid contractions or MVCs. To assess neural drive at MVC plateau, the amplitudes of the superimposed doublets were used to determine voluntary activation (VA) using the following formula:

$$\mathrm{VA} \left(\%\right)= \left[1- \left(\frac{{D}_{\mathrm{sup}}}{{D}_{\mathrm{con}}}\right)\right]*100,$$

where *D*_sup_ represents the superimposed doublet amplitude and *D*_con_ the potentiated doublet amplitude evoked at rest after the MVC. VA was calculated from one of the superimposed doublets (whichever was delivered at the greatest torque value) during the same MVC. Neural drive was also assessed from the RMS amplitude over a 500 ms epoch surrounding MVT (250 ms either side, without influence of artefact from electrical stimulation), normalised to *M*_max_, and the mean averaged across the three EMG sites to give a value for the whole quadriceps muscle (EMG_MVT_).

### Statistical analyses

All data were assessed for, and met, parametric assumptions prior to analysis. Descriptive data are reported as mean ± SD for *n* = 10. It was not possible to obtain values past 100 ms during the explosive voluntary contractions for two participants. This was because they were unable to perform the rapid voluntary contractions for the minimum required 1 s duration in enough voluntary efforts, whilst hyperthermic, often beginning to relax the muscle by 150 ms. Therefore, for *T*_150,_ RTD_100–150_, and EMG_0-150_ data are for *n* = 8. A Two-way repeated measures ANOVA was used to assess the influence of experimental trial (3 trials: pre- [Trial 1]; post-5 days [Trial 2]; and post-10 days [Trial 3]), at two different *T*_re_ (37 °C and 39 °C) on all physiological, perceptual, and neuromuscular dependant variables measured during the neuromuscular set. A one-way repeated measures ANOVA was used to assess responses within the HA sessions at HA 1, HA 5, and HA 10. Violations of sphericity were corrected using the Greenhouse–Geisser adjustment, when appropriate. Following a significant *F* value, pairwise differences were identified using stepwise Bonferroni-corrected paired *T* tests. Effect sizes for paired comparisons were calculated using Hedge’s *g* and interpreted as small (0.2), medium (0.5) or large (0.8) (Cohen 1988). The alpha level was set at *P* < 0.05. Statistical analysis was completed using SPSS version 26 (SPSS Inc., Chicago, IL).

## Results

### Responses within the HA sessions

Resting *T*_re_ (*P* = 0.010), resting HR (*P* = 0.003), sessional sweat rate (*P* = 0.002), mean session *T*_re_ (*P* = 0.003) and mean session HR (*P* = 0.043) all demonstrated overall improvements as the number of HA sessions increased. Post hoc analysis revealed midway through at HA 5, sweat rate increased (*P* = 0.024; *g* = 0.5), while mean session *T*_re_ (*P* = 0.015; *g* = 0.8) and mean session HR (*P* = 0.039; *g* = 0.6) reduced compared to HA 1. Resting *T*_re_ (*P* = 0.148; *g* = 0.6) and resting HR (*P* = 0.222; *g* = 0.6) were not statistically different at HA 5 from HA 1. There were no differences observed for time spent ≥ *T*_re_ 38.5 °C (*P* = 0.404) or ≥ *T*_re_ 39 °C (*P* = 0.795). HA 10 compared to HA 1 showed resting *T*_re_ (*P* = 0.012; *g* = 1.0), resting HR (*P* = 0.026; *g* = 1.2), and mean session *T*_re_ (*P* = 0.026; *g* = 0.9) decreased, while sweat rate increased (*P* = 0.021; *g* = 0.6), providing evidence of successful heat acclimation. Data are presented in Table 1.

**Table 1:** Thermoregulatory and cardiovascular responses within heat acclimation (HA) at baseline (HA 1), day 5 (HA 5) and day 10 of HA (HA 10)

Parameter	HA 1	HA 5	HA 10
Resting *T*_re_ (°C)	37.0 ± 0.4	36.7 ± 0.5	36.5 ± 0.5*
Resting HR (beat·min^−1^)	78 ± 8	2 ± 10	68 ± 7 *
Sweat rate (L·h^−1^)	1.5 ± 0.7	1.9 ± 0.7*	2.1 ± 0.9*
Mean session *T*_re_ (°C)	38.8 ± 0.3	38.5 ± 0.4*	38.4 ± 0.3*
Mean session HR	119 ± 12	110 ± 11*	113 ± 4
Duration *T*_re_ ≥ 38.5 °C (min)	59 ± 5	55 ± 15	59 ± 8
Duration *T*_re_ ≥ 39 °C (min)	40 ± 20	42 ± 24	36 ± 20

Data are means ± SD for *n* = 9

Post hoc significant difference from HA 1 (*P* < 0.05) is denoted by *

Rectal temperature (*T*_re_), heart rate (HR)

### Physiological and perceptual strain during the neuromuscular set

Participants were at the desired *T*_re_ during all the neuromuscular sets, with no main effects of trial (*P* = 0.353) or interaction (*P* = 0.629), but only an effect of *T*_re_ (*P* < 0.001), the latter imposed by study design. There was no main effect of trial on $\overline{T}$_sk_ (*P* = 0.267), but there was an effect of *T*_re_ (*P* < 0.001), in addition to an interaction effect (*P* = 0.004). Post hoc analysis revealed at *T*_re_ 37 °C, $\overline{T}$_sk_ was greater in Trial 3 than Trial 1 (*P* = 0.028; *g* = 1.0), but it was not statistically different in other between trial comparisons (*P* ≥ 0.194; *g* = 0.1–0.7). HR was affected by trial (*P* = 0.010), *T*_re_ (*P* < 0.001) and there was an interaction effect (*P* = 0.011). Post hoc analysis showed at *T*_re_ 37 °C, HR had increased in Trial 3 compared to Trial 1 (*P* = 0.019; *g* = 0.9), likely because some participants had to spend time in the heat passively warming to attain *T*_re_ ~ 37 °C before commencing neuromuscular set 1. While at *T*_re_ 39 °C HR had decreased in Trial 3 compared to Trial 2 (*P* = 0.036; *g* = 0.4). Other between trial comparisons were not statistically different (*P* ≥ 0.143; *g* = 0.1–0.6). No main effects of trial (*P* ≥ 0.147) or interaction (*P* ≥ 0.062) were observed for TS and TC. However, participants did feel hotter and more uncomfortable at *T*_re_ 39 °C compared to 37 °C in all trials (main effect of *T*_re_; *P* < 0.001). Data are presented in Table 2.

**Table 2:** Rectal temperature (*T*_re_), mean weighted skin temperature ($\overline{T}$_sk_), heart rate (HR), thermal sensation (TS) and thermal comfort (TC) during the neuromuscular sets of each trial

Parameter	Trial 1		Trial 2		Trial 3	
	*T*_re_ 37	*T*_re_ 39	*T*_re_ 37	*T*_re_ 39	*T*_re_ 37	*T*_re_ 39
*T*_re_ (°C)	37.1 ± 0.2	39.1 ± 0.2†††	37.0 ± 0.1	39.1 ± 0.2†††	37.0 ± 0.2	39.0 ± 0.1†††
$\overline{T}$_sk_ (°C)	35.7 ± 1.8	39.4 ± 0.7†††	36.4 ± 1.8	39.1 ± 0.8††	37.1 ± 0.7#	39.0 ± 0.5†††
HR (beat·min^−1^)	92 ± 19	136 ± 12†††	98 ± 22	144 ± 13†††	110 ± 18#	137 ± 18††‡
TS	5.4 ± 0.8	7.6 ± 0.8†††	5.6 ± 1.1	7.1 ± 1.0††	5.5 ± 0.9	7.2 ± 0.9†††
TC	2 ± 1	4 ± 1†††	2 ± 1	3 ± 1††	2 ± 1	3 ± 1††

Participants performed the same neuromuscular set at two different *T*_re_: 37 °C and 39 °C on three separate trial days pre- (Trial 1), post-5 days (Trial 2) and post-10 days (Trial 3) of heat acclimation. Measurements were taken at the start and the end of the set and averaged to give a mean value for each dependant variable

Data are means ± SD for *n* = 10

Post hoc significant difference is denoted by:

^†^(*P* < 0.05)

^††^(*P* < 0.005)

^†††^(*P* < 0.001), different from 37 °C

^#^(*P* < 0.05), different from Trial 1

^‡^(*P* < 0.05), different from Trial 2

### Voluntary torque and RTD

**Fig. 2:**
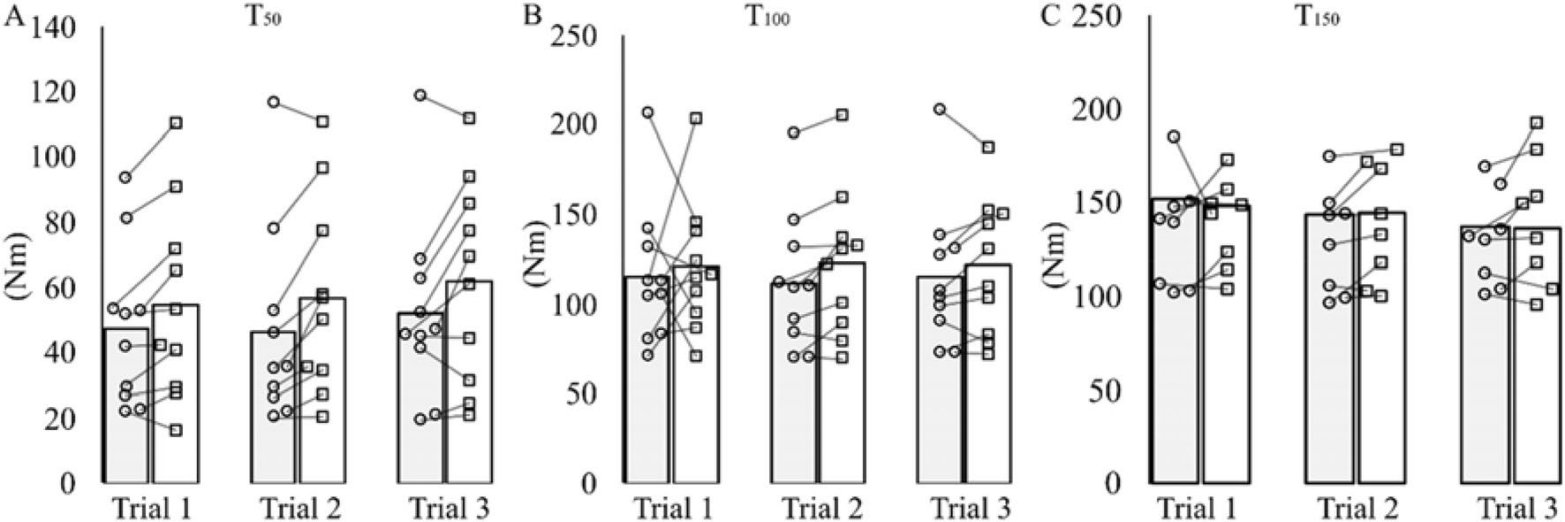
Rapid voluntary torque at; 50 ms (*T*_50_; **A**), 100 ms (*T*_100_; **B**) and 150 ms (*T*_150_; **C**) from contraction onset. Measurements were taken at two different rectal temperatures: 37 °C (grey bars and open circles) and 39 °C (open bars and open squares), and pre- (Trial 1), post-5 days (Trial 2) and post-10 days (Trial 3) of heat acclimation. Individual data points and group means presented for *n* = 10 at *T*_50_ and *T*_100_. Data are *n* = 8 for *T*_150_ because it was not possible to obtain torque values after 100 ms for two participants. For clarity, error bars have been omitted

**Fig. 3:**
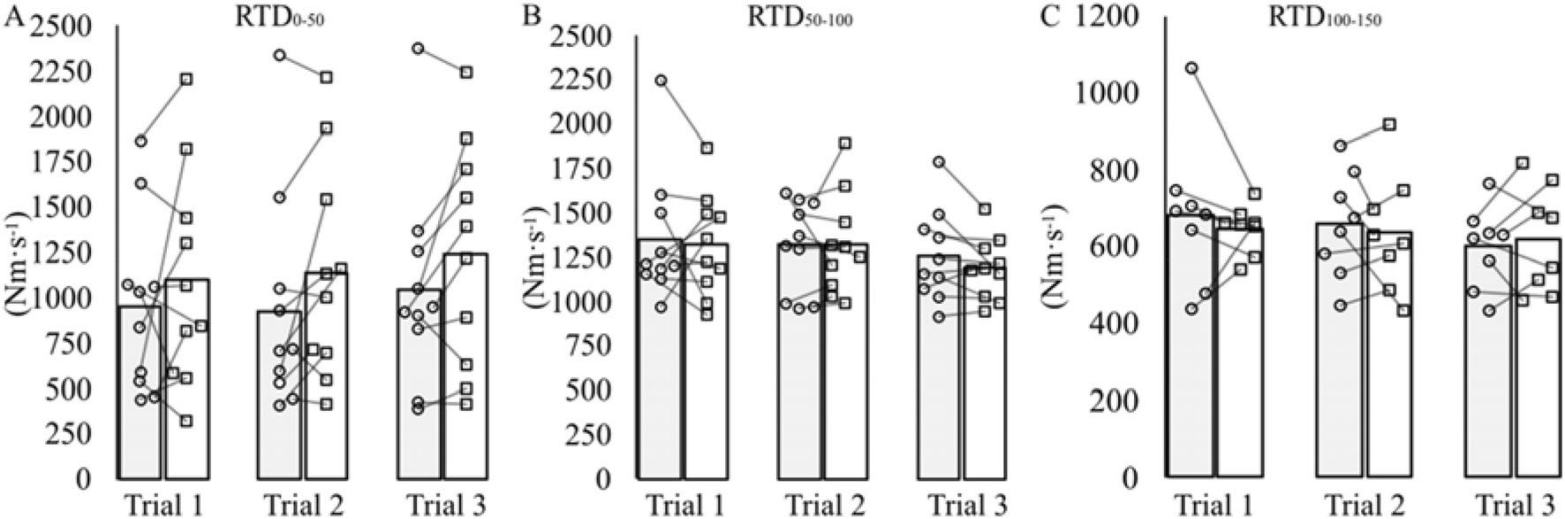
Rate of torque development at sequential time epochs, 0–50 ms (RTD_0–50_; **A**), 50–100 ms (RTD_50–100_; **B**) and 100–150 ms (RTD_100–150_; **C**). Measurements were taken at two different rectal temperatures: 37 °C (grey bars and open circles) and 39 °C (open bars and open squares), and pre- (Trial 1), post-5 days (Trial 2) and post-10 days (Trial 3) of heat acclimation. Individual data points and group means presented for *n* = 10 at RTD_0–50_ and RTD_50–100_. Data are n = 8 for RTD_100–150_ because it was not possible to obtain RTD values after 100 ms for two participants. For clarity, error bars have been omitted

MVT was not affected by experimental trial (*P* = 0.928), *T*_re_ (*P* = 0.524) or interaction (*P* = 0.653) (Table 3). Rapid torque production (*T*_50_, *T*_100_, and *T*_150_; Fig. 2) and voluntary RTD (RTD_0–50_, RTD_50–100_, and RTD_100–150_; Fig. 3) were also all unaffected by trial (*P* ≥ 0.064), T_re_ (*P* ≥ 0.071), or interaction (*P* ≥ 0.493).

**Table 3:** Maximum voluntary torque (MVT), surface EMG RMS at MVT (EMG_MVT_) normalised to *M*_max_, and voluntary activation (VA)

Parameter	Trial 1		Trial 2		Trial 3	
	*T*_re_ 37	*T*_re_ 39	*T*_re_ 37	*T*_re_ 39	*T*_re_ 37	*T*_re_ 39
MVT (Nm)	203 ± 57	197 ± 53	202 ± 59	201 ± 52	200 ± 52	202 ± 55
EMG_MVT_ (%)	7.3 ± 2.2	4.1 ± 1.7†††	6.6 ± 2.4	4.5 ± 1.1†	7.5 ± 2.7	4.8 ± 2.1†
VA (%)	92 ± 7	87 ± 13	88 ± 13	80 ± 18	89 ± 9	85 ± 14

Participants performed the same neuromuscular set at two different rectal temperatures (*T*_re_): 37 °C and 39 °C on three separate trial days pre- (Trial 1), post-5 days (Trial 2) and post-10 days (Trial 3) of heat acclimation.

Data are means ± SD for *n* = 10. Post hoc significant difference is denoted by:

^†^(*P* < 0.05)

^†††^(*P* < 0.001), different from 37 °C

### Neural drive

EMG_MVT_ (Table 3) decreased with the rise in *T*_re_ (*P* = 0.001), but was not affected by experimental trial (*P* = 0.509), or an interaction effect (*P* = 0.564). Post hoc analysis revealed at *T*_re_ 39 °C EMG_MVT_ had decreased compared to *T*_re_ 37 °C in all three trials (*P* ≤ 0. 021; *g* = 1.1–1.6). There were no main effects of HA (*P* = 0.146), *T*_re_ (*P* = 0.060), or interaction effect (*P* = 0.790) for VA (Table 3).

**Fig. 4:**
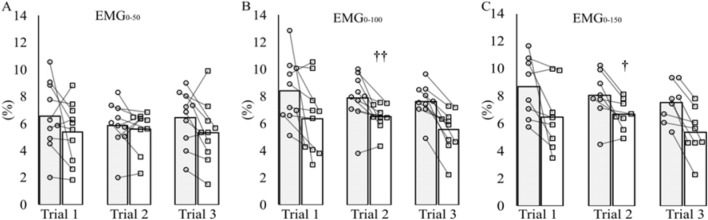
Surface EMG RMS normalised to *M*_max_ at 0–50 ms (EMG_0–50_; **A**), 0–100 ms (EMG_0–100_; **B**) and 0–150 ms (EMG_0–150_; **C**). Measurements were taken at two different rectal temperatures: 37 °C (grey bars and open circles) and 39 °C (open bars and open squares), and pre- (Trial 1), post-5 days (Trial 2) and post-10 days (Trial 3) of heat acclimation. Individual data points and group means presented for *n* = 10 at EMG_0–50_ and EMG_0–100_. Data are *n* = 8 for EMG_0–150_ because it was not possible to obtain EMG values after 100 ms for two participants. For clarity error bars have been omitted. Post hoc significant difference is denoted by: † (*P* < 0.05), †† (< 0.005), different from 37 °C

No main effect of trial (*P* = 0.816), *T*_re_ (*P* = 0.101), or interaction (*P* = 0.097) was observed for EMG_0–50_ (Fig. 4A). No effects of trial (*P* ≥ 0.467) or interaction (*P* ≥ 0.326) were observed for EMG_0–100_ (Fig. 4B) or EMG_0–150_ (Fig. 4C), but the rise in *T*_re_ did result in a decrease (*P* ≤ 0.016) in both these variables. Post hoc analysis revealed at *T*_re_ 39 °C EMG_0–100_ had decreased compared to *T*_re_ 37 °C in Trial 2 (*P* = 0.003; *g* = 0.9), but was not statistically different in the other trials (*P* ≥ 0.051; *g* = 0.8–1.4). EMG_0–150_ also decreased at *T*_re_ 39 °C compared to *T*_re_ 37 °C in Trial 2 (*P* = 0.020; *g* = 0.8), but was not statistically different in Trial 1 or 3 (*P* ≥ 0.055; *g* = 0.9–1.3).

### Intrinsic contractile properties

No main effect of experimental trial was observed on the twitch dependant variables (*P* ≥ 0. 116). There was an interaction effect for ½ RT (*P* = 0.032), but not for any other parameter (*P* ≥ 0. 155). There was a main effect of *T*_re,_ and the increase in *T*_re_ caused greater (PT, RTD_0–50_, pRTD) and faster (TPT and ½ RT) twitch responses (*P* ≤ 0.027). Post hoc analysis revealed at *T*_re_ 39 °C twitch PT had increased compared to *T*_re_ 37 °C in Trial 1 and 2 (*P* ≤ 0.037, *g* = 0.5), but was not statistically different in Trial 3 (*P* = 0.059; *g* = 0.3). Twitch RTD_0–50_ and pRTD were greater at *T*_re_ 39 °C in all trials (*P* ≤ 0.013; *g* = 0.4–0.7). Twitch TPT was faster at *T*_re_ 39 °C compared to *T*_re_ 37 °C in Trial 1 and Trial 2 (*P* ≤ 0.018; *g* = 1.5), but not in Trial 3 (*P* = 0.133; *g* = 0.9). Twitch ½ RT was also faster at *T*_re_ 39 °C in Trial 1 (*P* = 0.002; *g* = 1.3), but not statistically different in the other trials (*P* ≥ 0.098; *g* = 0.6). Data are presented in Table 4.

**Table 4:** Evoked peak torque (PT), rate of torque development during initial 50 ms from contraction onset (RTD_0–50_), peak rate of torque development (pRTD), time to peak torque (TPT) and half-relaxation time (½ RT) during supramaximal twitch and octet contractions

Parameter	Trial 1		Trial 2		Trial 3	
	*T*_re_ 37	*T*_re_ 39	*T*_re_ 37	*T*_re_ 39	*T*_re_ 37	*T*_re_ 39
Twitch						
PT (Nm)	31 ± 12	39 ± 20†	29 ± 12	39 ± 22†	36 ± 17	43 ± 26
RTD_0–50_ (Nm·s^−1^)	405 ± 218	615 ± 348††	369 ± 253	626 ± 392††	500 ± 304	674 ± 421††
pRTD (Nm·s^−1^)	863 ± 405	1234 ± 610††	825 ± 414	1246 ± 687††	1010 ± 593	1361 ± 888†
TPT (ms)	94 ± 12	76 ± 11†	93 ± 8	77 ± 11†	92 ± 9	84 ± 8
½ RT (ms)	97 ± 32	65 ± 11††	81 ± 13	72 ± 15	84 ± 14	72 ± 23
Octet						
PT (Nm)	124 ± 51	137 ± 57	128 ± 58	135 ± 61	131 ± 53	145 ± 62††
RTD_0–50_ (Nm·s^−1^)	1372 ± 617	1775 ± 670††	1378 ± 714	1671 ± 685†††	1480 ± 668	1744 ± 699†††
pRTD (Nm·s^−1^)	2651 ± 1469	3326 ± 1373††	2426 ± 1143	3275 ± 1575††	2940 ± 1368	3582 ± 1638††
TPT (ms)	147 ± 10	127 ± 7†††	147 ± 12	133 ± 7††	149 ± 11	136 ± 11††
½ RT (ms)	78 ± 14	58 ± 18†	79 ± 13	58 ± 18††	83 ± 15	65 ± 11†

Participants performed the same neuromuscular set at two different rectal temperatures (T_re_): 37 °C and 39 °C on three separate trial days pre- (Trial 1), post-5 days (Trial 2) and post-10 days (Trial 3) of heat acclimation

Data are means ± SD for *n* = 10

Post hoc significant difference is denoted by:

^†^(*P* < 0.05)

^††^(*P* < 0.005)

^†††^(*P* < 0.001), different from 37 °C

No main effect of experimental trial (*P* ≥ 0.059) or interaction effects (*P* ≥ 0. 062) were observed on the octet dependant variables. A main effect and rise in *T*_re_ caused greater (PT, RTD_0–50_, pRTD) and faster (TPT and ½ RT) octet responses (*P* ≤ 0.019). Post hoc analysis revealed at *T*_re_ 39 °C octet PT had increased compared to *T*_re_ 37 °C in trial 3 (*P* = 0.004, *g* = 0.2), but was not statistically different in the other trials (*P* ≥ 0.082; *g* = 0.1–0.2). Octet RTD_0–50_ and pRTD were greater at *T*_re_ 39 °C in all trials (*P* ≤ 0.002; *g* = 0.4–0.6), while octet TPT and ½ RT were faster at *T*_re_ 39 °C in all trials (*P* ≤ 0.044; *g* = 0.9–2.2). Data are presented in Table 4.

## Discussion

This study investigated the effect of 5 and 10 days of isothermal HA on the neural and contractile mechanisms that determine rapid and maximal torque capacity, whilst normothermic and hyperthermic. Consistent with our previous observations (Gordon et al. 2021), acute hyperthermia (independent of HA) reduced neural drive at MVT and during rapid voluntary contractions. The reductions in neural drive did not reduce MVT or RTD, potentially due to warmer muscles increasing intrinsic contractile (twitch and octet) torques and the speed of torque development (Table 4). HA induced favourable physiological adaptations to the heat after 10 days of heat exposure, with successful manipulation of the “classic” markers of HA (e.g. reduction in resting *T*_re_ and HR and an increased sweat rate). However, there was no evidence that HA, independent of hyperthermia, improved any measured aspect of neuromuscular function, nor did HA attenuate the observed hyperthermia-induced reductions in neural drive. Nevertheless, there was an interaction effect on ½ RT, in which the reduction in ½ RT caused by hyperthermia in Trial 1 was not observable in Trial 2 or 3. Speculatively, this preservation of ½ RT when the muscle is hot, following HA, may have functional benefits for exercise in the heat, as the faster ½ RT typically observed with warmer muscles is thought to negatively affect twitch force summation, necessitating a greater neural drive to obtain the same absolute force (Todd et al. 2005; Périard et al. 2014b).

### Effects of acute hyperthermia

During the experimental trial sessions, heat exposure increased all thermoregulatory, cardiovascular, and perceptual responses, while *T*_re_ was successfully clamped across the different trials at pre-, post-5 days, and post-10 days of HA (Table 2). Similarly, subjective perceptual ratings of the heat (TS) and comfort (TC) were not altered by HA, with participants feeling “very hot” and “uncomfortable” in all trials. Taken together, these data demonstrate that the neuromuscular set was performed under similar levels of actual and perceived thermal strain.

MVT (Table 3) was not affected by hyperthermia. Despite no change in MVT, neural drive did decrease with hyperthermia, evidenced by lower EMG_MVT_ (main effect of *T*_re_; *P* < 0.05; Table 3). The reduction in neural drive at MVC plateau with acute hyperthermia corroborates previous research from our laboratory which used the same neuromuscular assessment protocol (Gordon et al. 2021). However, our previous study (Gordon et al. 2021) found MVT to decrease concomitantly with neural drive due to acute hyperthermia. It is unclear why MVT did not decrease with hyperthermia in the current study, but it is possibly linked to the hyperthermia-induced increased torque capacity of the muscle, evidenced by the acute increases in twitch and octet PT (Table 4). Specifically, the increased torque capacity may have countered a reduction in neural drive, maintaining MVT. Furthermore, the training status of participants used in this study (endurance trained amateur athletes) and that of Gordon et al. (2021) (recreationally trained males) was different and data suggests that more aerobically trained individuals are better able to tolerate high heat stress, leading to smaller performance decrements in the heat (Cheung and McLellan 1998; Guy et al. 2015). Additionally, closer post hoc inspection of the individual data revealed a speculative effect of sex on neuromuscular function during acute hyperthermia. Specifically, in Trial 1, acute hyperthermia caused a 6.2% decline in MVT in the males (paired *t* test; *P* = 0.205; *g* = 0.6), but only a 1.1% decline in females (paired *t* test; *P* = 0.765; *g* = 0.1). Thus, males may be more susceptible to declines in MVT than females which if true, would reduce the chances of observing significant declines in MVT across a mixed-sex sample. Our previous study (Gordon et al. 2021) and that of others who observed reduced MVT with hyperthermia, have included only male participants (Morrison et al. 2004; Périard et al. 2014a), which might explain why their results contrast with ours. On the other hand, other studies have used mixed male and female cohorts and observed reduced MVT with hyperthermia (Todd et al. 2005; Thomas et al. 2006), but did not assess the effects of sex. The current study is under-powered for considering between-sex differences and so we recommend future research considers the effect of sex on neuromuscular responses to hyperthermia.

Similar to MVT, voluntary RTD was unaffected by acute hyperthermia, despite a reduction in neural drive evidenced by a main effect for decreased EMG_0–100_ (Fig. 4B) and EMG_0–150_ (Fig. 4C), which is consistent with what we observed in our previous study (Gordon et al. 2021). The preservation of rapid voluntary torque is likely linked to the faster contractile properties in hyperthermic conditions (Table 4), which increase the intrinsic capacity for RTD and counter the reduction in neural drive. The faster contractile properties with hyperthermia are likely due to increased muscle temperature. Although muscle temperature was not measured in the present study, research reporting similar magnitudes of change in T_re_ to the present study when heating the whole body have observed concomitant increases in muscle temperature. The muscle temperatures were reported from resting pre-heating values of ~ 35 °C (*T*_re_ ~ 37 °C) up to end of trial values of ~ 39.4 °C (*T*_re_ ~ 39.5 °C) (Périard et al. 2014b; Racinais and Girard, 2012). Greater muscle temperatures improve muscle fibre conduction velocity (Gray et al. 2006; Farina et al. 2005) and excitation contraction coupling (Brody 1976). This is due to the faster rate of myosin–actin attachment during cross-bridge cycling (Davies et al. 1982) and ATPase activity (Bárány 1967; Stein et al. 1982). Additionally, elevated core temperature appears to shorten the electromechanical delay between muscle activation and force production onsets, reflecting the faster excitation contraction coupling (Gordon et al. 2021). These electromechanical mechanisms observed with increased muscle temperature would contribute to the faster contractile responses observed in the present study. Our data align with others that have found faster twitch (Gordon et al. 2021; Périard et al. 2014b) and octet ½ RT (Gordon et al. 2021) during moderate (*T*_re_ 38.5 °C) and severe (*T*_re_ 39.5 °C) hyperthermia.

### Effects of HA

The HA protocol employed in the present study successfully induced adaptation to repeated heat exposures (Table 1). Sweat rate was greater at HA 5 (+ 0.4 L·h^−1^), whilst reductions in resting *T*_re_ (− 0.5 °C) and HR (− 10 beat·min^−1^) and an increase in the sweating rate (+ 0.6 L·h^−1^) were observed by HA 10 relative to HA 1, the magnitude of which are all consistent with the HA literature (Sawka et al. 2011; Tyler et al. 2016). These data provide evidence that participants improved heat storage capacity, lowered cardiovascular strain, and subsequently ameliorated the heat loss mechanisms for more efficient thermoregulation.

We hypothesised that a regime of HA would attenuate the expected hyperthermia-induced reduction in neural drive, and thus attenuate a decline in MVT and enable faster RTD due to the faster contractile properties of the muscle. However, HA did not attenuate reductions in neural drive whilst hyperthermic, and in agreement with others who found no influence centrally mediated mechanisms (Wingfield et al. 2016; Osborne et al. 2021; Brazaitis and Skurvydas 2010). It is therefore not surprising that the effects (or lack thereof) of acute hyperthermia on MVT and rapid voluntary torque were unchanged following HA. To the author’s knowledge, the effect of 10 days of HA on rapid voluntary torque has not previously been investigated. However, our findings on MVT contrast with others. Racinais et al. (2017b) reported plantar flexion MVC torque in hyperthermic conditions to be 16% (*P* < 0.05) greater after 11 days of passive HA. Osborne et al. (2021) similarly observed increased knee extension torque (*P* < 0.05) in hyperthermic conditions following 5 days of HA. Nevertheless, the above increases in MVC torque whilst hyperthermic, following HA, were not attributed to increased neural drive to the muscle. We therefore speculate that for constant levels of hyperthermia and thermal sensation (as observed in this study; Table 2), reduced neural drive during whole-body hyperthermia is inevitable, and this effect cannot be ameliorated through HA.

Independent of hyperthermia, we did not observe any change in MVT (Table 3), RTD (Figs. 2, 3, 4), or any other neuromuscular variable (Table 4), following 5 or 10 days of HA. In contrast Racinais et al. (2017b) reported an 11% increase (*P* < 0.05) in maximal plantar flexion MVT in temperate (24 °C) conditions, following 11 days of passive HA. The increased baseline MVT was not due to changes in neural drive which remained unchanged following HA. Instead, the same study (Racinais Wilson and Périard 2017b) observed increased twitch peak torque in in temperate conditions following HA and suggested this HA-induced improvement in intrinsic contractile function likely caused the improvement in MVT. In contrast, our data showed the twitch and octet responses in temperate conditions (independent of hyperthermia) were unaffected by HA, which may explain why we did not observe improved MVT in cool conditions, whereas Racinais et al. (2017b) did. In addition to increased peak twitch torque, the same study (Racinais et al. 2017b) did observe a slowing of twitch ½ RT (*P* < 0.05) during the controlled background muscle contraction at 10% of MVC. Whilst our current study did not find evidence of slower ½ RT in baseline temperate conditions following HA, we did observe an interaction effect (*P* < 0.05) of acute hyperthermia and HA on twitch ½ RT (Table 4), providing some supporting evidence that HA affects the intrinsic contractile properties of muscle.

To the author’s knowledge, the interaction effect of hyperthermia and HA on twitch ½ RT (caused by an attenuation of the hyperthermia-induced reduction of ½ RT following HA) is a novel finding (Table 4). The mechanism and implication of this effect are unclear. Half RT is a measure of skeletal muscle relaxation after a single twitch or tetanic contraction initiated by reductions in Ca^2+^ concentration in the sarcoplasmic reticulum. The efficiency of this process is modulated by the dissociation of Ca^2+^ from troponin, translocation of the Ca^2+^ to the sarcoplasmic reticulum and then its subsequent active re-uptake (Bennett 1985). We speculate that the maintenance of ½ RT whilst hyperthermic post-HA may indicate phenotypic changes to the release and re-uptake of Ca^2+^. Attenuating the hyperthermic-induced reduction of ½ RT may serve to benefit force summation, by minimising the rightward shift in the force–frequency relationship that occurs with elevated core, and in turn muscle temperature (Périard et al. 2014b; Todd et al. 2005). The residual effect could be to help maintain MVT during whole-body hyperthermia.

The authors recognise some limitations of the study. The study design attempted to clamp *T*_re_ to standardise when the neuromuscular measurements were completed, which in Trial 3 led to elevated $\overline{T}$_sk_ and HR values when baseline values were measured (Table 2). This is because successful HA lowered resting *T*_re_, meaning participants spent longer time passively heating prior to the first neuromuscular set to attain a *T*_re_ of 37 °C. Elevated HR and $\overline{T}$_sk_ (due to cardiovascular drift) suggest greater cardiovascular strain (Rowell 1974). However, because of the passive nature of the heating protocol used, the influence of moderately increased cardiovascular strain was likely to have been minimal. In addition, voluntary and involuntary evoked torque responses were similar at *T*_re_ of 37 °C pre- and post-HA, suggesting despite raised cardiovascular strain and $\overline{T}$_sk,_ this did not affect neuromuscular responses.

## Conclusion

In conclusion, neural drive was reduced during both maximal and rapid voluntary contractions during hyperthermia, but this did not affect MVT or rapid voluntary torque production. The preservation of voluntary torque when hyperthermic is likely due to improvements in contractile function mitigating the decline in neural drive. Neither 5 nor 10 days of HA mitigated the hyperthermia-induced decline in neural drive. Similarly, HA exhibited no beneficial effects on measures of voluntary torque production, independent of acute hyperthermia. Nevertheless, a novel finding from this study was the interaction between hyperthermia and HA on twitch ½ RT, which reduced the hyperthermia-induced speeding-up of rate of muscle relaxation, following HA. This could theoretically benefit force summation when exercising in the heat.

## Data Availability

The datasets generated and analysed during the current study are available from the corresponding author on reasonable request.

## Abbreviations

$\overline{T}$_s__k_: Mean weighted skin temperature (°C)
½ RT: Half-relaxation time (ms)
ANOVA: Analysis of variance
*g*: Hedge’s *g* effect size
EMG: Electromyography
HA: Heat acclimation
HR: Heart rate (beat·min^−^^1^)
MVC: Maximal voluntary contraction
MVT: Maximal voluntary torque (Nm)
pRTD: Peak rate of torque development
PT: Peak torque (Nm)
Rh: Relative humidity
RTD: Rate of torque development (Nm·s^−^^1^)
TC: Thermal comfort
TPT: Time to peak torque (ms)
T_re_: Rectal temperature (°C)
TS: Thermal sensation
VA: Voluntary activation (%)
